# Topical Tenofovir Pre-exposure Prophylaxis and Mucosal HIV-Specific Fc-Mediated Antibody Activities in Women

**DOI:** 10.3389/fimmu.2020.01274

**Published:** 2020-07-06

**Authors:** Kimone Leigh Fisher, Jennifer M. Mabuka, Aida Sivro, Sinaye Ngcapu, Jo-Ann Shelley Passmore, Farzana Osman, Bongiwe Ndlovu, Quarraisha Abdool Karim, Salim S. Abdool Karim, Amy W. Chung, Cheryl Baxter, Derseree Archary

**Affiliations:** ^1^Centre for the AIDS Programme of Research in South Africa, University of KwaZulu-Natal, Durban, South Africa; ^2^Africa Health Research Institute, University of KwaZulu-Natal, Durban, South Africa; ^3^HIV Pathogenesis Programme, The Doris Duke Medical Research Institute, Nelson R. Mandela School of Medicine, University of KwaZulu-Natal, Durban, South Africa; ^4^Department of Medical Microbiology, University of KwaZulu-Natal, Durban, South Africa; ^5^Institute of Infectious Diseases and Molecular Medicine (IDM), University of Cape Town, and National Health Laboratory Service, Cape Town, South Africa; ^6^Department of Epidemiology, Mailman School of Public Health, Columbia University, New York, NY, United States; ^7^Department of Microbiology and Immunology, The Peter Doherty Institute for Infection and Immunity, University of Melbourne, Melbourne, VIC, Australia; ^8^Department of Public Health, University of KwaZulu-Natal, Durban, South Africa

**Keywords:** Fc-mediated activity, tenofovir, HIV, women, ADCC–antibody dependent cellular cytotoxicity, ADNP, genital tract

## Abstract

The RV144 HIV-vaccine trial highlighted the importance of envelope-specific non-neutralizing antibody (nNAb) Fc-mediated functions as immune correlates of reduced risk of infection. Since pre-exposure prophylaxis (PrEP) and HIV-vaccines are being used as a combination prevention strategy in at risk populations, the effects of PrEP on nNAb functions both mucosally and systemically remain undefined. Previous animal and human studies demonstrated reduced HIV-specific antibody binding avidity post-HIV seroconversion with PrEP, which in turn may affect antibody functionality. In seroconverters from the CAPRISA 004 tenofovir gel trial, we previously reported significantly higher detection and titres of HIV-specific binding antibodies in the plasma and genital tract (GT) that distinguished the tenofovir from the placebo arm. We hypothesized that higher HIV-specific antibody titres and detection reflected corresponding increased antibody-dependent neutrophil-mediated phagocytosis (ADNP) and NK-cell-activated antibody-dependent cellular cytotoxic (ADCC) activities. HIV-specific V1V2-gp70, gp120, gp41, p66, and p24 antibodies in GT and plasma samples of 48 seroconverters from the CAPRISA 004 tenofovir gel trial were tested for ADCP and ADCC at 3, 6- and 12-months post-HIV-infection. GT gp41- and p24-specific ADNP were significantly higher in the tenofovir than the placebo arm at 6 and 12 months respectively (*p* < 0.05). Plasma gp120-, gp41-, and p66-specific ADNP, and GT gp41-specific ADCC increased significantly over time (*p* < 0.05) in the tenofovir arm. In the tenofovir arm only, significant inverse correlations were observed between gp120-specific ADCC and gp120-antibody titres (*r* = −0.54; *p* = 0.009), and gp41-specific ADNP and gp41-specific antibody titres at 6 months post-infection (*r* = −0.50; *p* = 0.015). In addition, in the tenofovir arm, gp41-specific ADCC showed significant direct correlations between the compartments (*r* = 0.53; *p* = 0.045). Certain HIV-specific nNAb activities not only dominate specific immunological compartments but can also exhibit diverse functions within the same compartment. Our previous findings of increased HIV specific antibody detection and titres in women who used tenofovir gel, and the limited differences in nNAb activities between the arms, suggest that prior PrEP did not modulate these nNAb functions post-HIV seroconversion. Together these data provide insight into envelope-specific-nNAb Fc-mediated functions at the site of exposure which may inform on ensuing immunity during combination HIV prevention strategies including PrEP and HIV vaccines.

## Introduction

HIV continues to be a major global health challenge. In particular, young women in Africa (aged 15–24 years) remain vulnerable to HIV infection (1). Despite the advent and testing of several biomedical prevention strategies such as intra-vaginal rings containing antiretroviral drugs (2), topical tenofovir gel as pre-exposure prophylaxis (PrEP) (3), or oral PrEP (4, 5) levels of protection among women have been inconsistent, ranging from −49 to 76% (3–5). These data therefore underscore the urgent need for an efficacious HIV vaccine. In the current era of prevention, where future combination HIV prevention strategies will likely include both PrEP and HIV vaccines especially in at-risk populations, elucidating the effects of prior use of PrEP on immunity are important.

A non-human primate (NHP) study of oral tenofovir reported that animals with breakthrough SHIV infection had reduced CD4^+^ T cell loss and lowered immune activation compared to control animals (6). Women who experienced breakthrough HIV infections and were assigned to the tenofovir gel arm in the CAPRISA 004 trial had delayed antibody binding avidity (7) and preserved Gag-specific CD4^+^ T cells (8), which may likely have aided B cells to produce p24-specific or other antibodies. P24-specific antibodies have been associated with improved CD4^+^ T cell counts and lower viral loads (9) and has also been associated with HIV control (10). In addition, p66 antibodies have been associated with a decreased rate of disease progression (11, 12) and Fc-mediated antiviral activities (13). In seroconverters from the CAPRISA tenofovir gel trial, we previously reported significantly higher detection and titres of gp120-IgG, p66-IgG, and gp41-IgA in the plasma and genital tracts of women in the tenofovir arm, compared to those in the placebo arm (14). Whether these antibodies were capable of binding to infected cells and/or exerting Fc-mediated antiviral activities remains unknown [reviewed by Overbaugh and Morris (15)].

The ultimate goal of a vaccine is to induce robust antibody responses capable of neutralizing a variety of HIV strains to prevent HIV infection. To date, the RV144 HIV-vaccine is the only trial to show a 31.2% protective efficacy (16). The analyses of the correlates of reduced risk of infection were attributed to the presence of HIV-specific non-neutralizing binding antibodies (nNAb) which elicited enhanced Fc-mediated NK-cell-activated antibody-dependent cellular cytotoxicity (ADCC) in the presence of limited tier 1 nNAbs (17), but in the absence of IgA (18). Even in preclinical macaque studies, Fc-mediated antiviral activities were shown to protect animals from SHIV infections (19–21). Furthermore, in passive immunity studies in macaques, broadly neutralizing antibodies such as PGT121, were shown to exert viral clearance using Fc-mediated antiviral activities in distal tissues (22). Human cohort studies have also underscored the role of ADCC (9, 13, 23, 24) and antibody-dependent neutrophil-mediated phagocytosis (ADNP) activities in ameliorating HIV disease progression (25). Antibodies capable of effecting ADCC in the breastmilk have been associated with significant reductions in mother-to-child-transmissions of HIV (26) and decreased infant mortality (27). Given that the genital tract is the most common site of sexual HIV transmission, the presence of functional mucosal antibodies may aid in preventing local infection or delaying/halting disease progression through viral control. Additional evidence reporting ADCC activities in the female genital tract and subsequent decreases in genital tract viral loads, emphasizes the importance of Fc-mediated antiviral activities at vulnerable sites of transmission (28, 29).

The effects of PrEP however, on HIV-specific antibody (IgG) Fc-mediated antiviral activities remain unknown. Based on our previous findings, we posited that the higher titres and detection of certain HIV-specific antibodies in women who used the tenofovir gel (14), translated to a corresponding increase in HIV-specific nNAb activities in the genital tract and plasma relative to those in the placebo arm. In this study, we sought to elucidate the effects of prior PrEP on nNAb functional immune responses in the genital tracts and plasma of women who experienced breakthrough HIV infections.

## Materials and Methods

### Study Population

This sub-study included 48 women who acquired HIV infection while participating in the CAPRISA 004 tenofovir gel trial (NCT00441298) (3). Twenty-four women from the tenofovir arm were case matched to 24 women in the placebo arm according to their viral loads (VL) and CD4^+^ T-cell counts at six-months post-infection. Therefore, there were no significant differences in either the CD4+ T cell counts or HIV viral loads between the arms. Plasma and cervicovaginal lavages (CVL) were collected as previously described (3). Plasma and CVL samples from 3, 6-, and 12-months post-infection were included in this sub-study. These women remained antiretroviral treatment naïve during this period of observation post-HIV infection. Tenofovir drug levels were previously measured in the CVL aspirates of women from the CAPRISA 004 trial (30), and these women were HIV uninfected at the time of the tenofovir drug measurements. Ethics approvals for the original study (E111/06) and for this sub-study (BE241/16) were obtained from the University of KwaZulu-Natal Biomedical Research Ethics Committee (BREC).

### Plasma IgG and CVL IgG Isolation and Quantification

Immunoglobulin (Ig) G from the plasma of women in both arms were positively selected using an IgG isolation kit (Thermofisher Scientific, Waltham, MA), as per the manufacturer's instructions. CVL samples for the mucosal experiments were used without further purifying out the IgGs, due to the limited volume of the CVLs available.

### Antibody Dependent Neutrophil Mediated Phagocytosis (ADNP)

**Figure 1:**
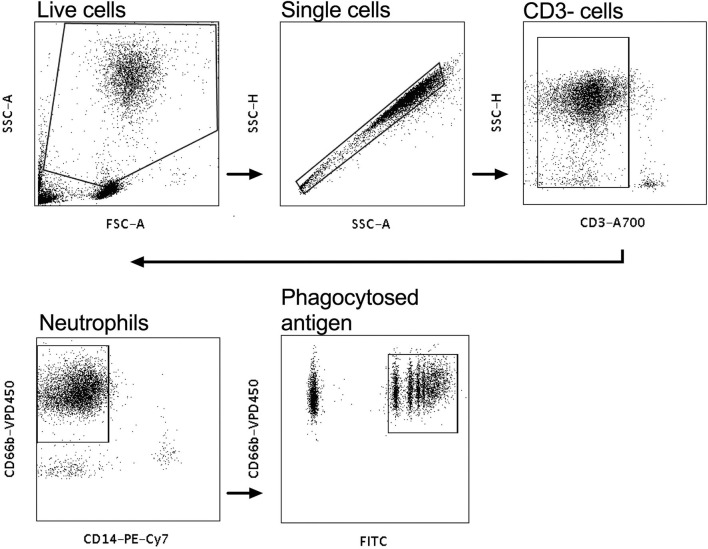
Gating strategy for antibody-dependent neutrophil phagocytic activity (ADNP). White blood cells were gated on, and the CD3^−^ population selected. CD14^−^, CD66^+^ populations were gated on to identify neutrophils and the CD66^+^FITC^+^ cells were gated on to identify phagocytosed neutravidin bound antigen beads.

Neutrophil mediated phagocytosis assays were performed as previously described (31). Briefly, white blood cells were isolated from fresh whole blood of healthy volunteers with ACK lysis buffer (Life Technologies, Grand Island, NY), as per the manufacturer's instructions and then resuspended to ~50,000 cells/mL in the volume of R10 media required for the assays. Neutrophils were then activated using isolated IgG and CVL from HIV infected women from both the tenofovir and placebo arms. A 100 μl of 0.50 mg/ml purified IgG from the plasma was used for these experiments and for the genital tract experiments 100 μl of CVL. Neutravidin beads were biotinylated to one of five HIV antigens: Con6 gp120/B (Consensus Group M gp120), gp70_B CaseA_V1_V2 (V1V2-gp70), gp41 (Ectodomain HIV-1), p66 RT (Immune Technology, California, USA or ImmunoDX, Massachusetts, USA), and p24 (HIV-1/Clade B/C CN54). The assays were conducted as previously described in (31) and the data were analyzed according to the protocol established by Darrah et al. (32). CD3 A700 was used to stain for CD3 negative cells, CD14 PE-Cy7, CD66b V450 (BD Biosciences, New Jersey, USA) were used to stain for neutrophils and the negative controls, which were the normal human serum (NHS) and phosphate buffered saline (PBS), respectively. Following staining, the samples were acquired on a LSR Fortessa (BD Biosciences, California, USA) as previously described (31). PBS and NHS were used to gate on CD3^−^, CD66^+^, CD14^−^ neutrophils that phagocytosed biotinylated antigen that was identified by the FITC^+^ population (Figure 1). Phagocytic scores were determined using an ADNP-specific gating strategy (Figure 1) and the integrated mean fluorescent intensity (MFI) with the following modification—% of neutrophils taking up beads x MFI/ 10,000 (32).

### NK-Cell Activated Antibody Dependent Cellular Cytotoxicity (ADCC)

**Figure 2:**
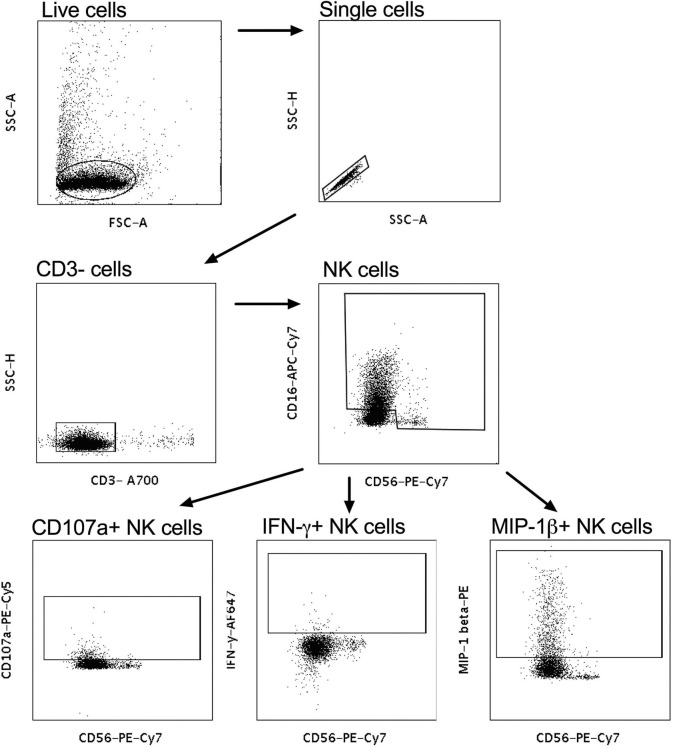
Gating strategy for NK-cell-activated antibody-dependent cellular cytotoxicity (ADCC). CD3^−^ cells were gated on and subsequently CD56^+^CD16^+^ NK cells were selected. CD56^+^CD107a^+^, CD56^+^IFN-γ^+^, and CD56^+^MIP-1β^+^ were used to identify cytotoxic markers and antiviral cytokines for activated NK cells.

Intracellular staining was used to assess the effector functions of activated natural killer (NK) cells isolated by negative selection (RosetteSep kit, Stemcell technologies) from fresh whole blood from healthy donors, as previously described (24), with the following modifications – 50 μl of 0.25 mg/ml IgG purified from plasma (data not shown) or 50 μl CVL were incubated with the one of four HIV antigens Con6 gp120/B (Consensus Group M gp120), gp41 (Ectodomain) (HIV-1), p66 RT, and p24 (HIV-1/Clade B/C CN54) (Immune Technology, California, USA or ImmunoDX, Massachusetts, USA) and 50,000 NK cells/well. Following incubation with monensin and Brefeldin A as well as CD107a, cells were stained with CD3^−^ A700, CD56 PE-Cy-7, CD16 APC-Cy7, IFN-γ APC, and MIP1-β PE (BD Biosciences, California, USA) and were then fixed with BD cell fix and then acquired on a LSR Fortessa (BD Biosciences, California, USA). Enriched NK cells were acquired and selected for singlets, followed by CD3 negative lymphocytes that were CD56^+^/CD16^+^ (Figure 2). The release of CD107a, IFN-γ and MIP-1β was used to assess frequency of activated NK cells to determine NK-cell activated ADCC (Figure 2) (9). When calculating HIV-specific antiviral activities, the non-specific ADCC activity obtained as background activity from PBS/NHS was subtracted from HIV-specific antibody-mediated ADCC activities.

### Statistical Analyses

Wilcoxon signed-rank test was used to compare differences in the plasma IgG and CVL phagoscores at different time points (i.e., 3–6 months, 6–12 months, and 3–12 months). Dunn's post hoc analyses could not be performed as there were limited matched sample availability at certain time points. Wilcoxon-Mann-Whitney test was used to compare two medians between tenofovir and placebo for %CD107a expression as a marker of NK cell ADCC activities, as well as %IFN-γ and %MIP-1β cytokine responses. No adjustments were made for multiple comparisons for either the ADNP or ADCC data. Spearman Rank correlation analyses were performed to determine inter-compartmental relationships, CD4^+^ T cell counts and VL in relation to Fc-mediated activities. Linear mixed models were used to analyse the change in ADNP and ADCC responses over time for each HIV protein and were adjusted for multiple comparisons using the false discovery rate method. Statistical analyses were performed using GraphPad Prism version 8 and SAS versions 9.4 (SAS Institute Inc., Cary) software.

## Results

### Demographics of Study Participants From the CAPRISA 004 Tenofovir Gel Trial

As previously reported in Archary et al. (14), the women in both the tenofovir and placebo arms were similar in terms of their age, level of education, numbers of sexual partners, condom use, use of hormonal contraception, and disease progression markers such as CD4^+^ T cell counts and viral loads at 6 months post-infection (Table 1). The only difference was that women in the tenofovir arm had a significant 5 month delay in time to HIV infection, compared to those in the placebo arm (p < 0.02) (14). In the tenofovir arm, 58.3% of the women had detectable tenofovir drug levels in the CVL aspirates.

**Table 1:** Demographics and clinical characteristics of the sub-set of women who acquire HIV infections during the CAPRISA 004 trials (*n* = 48).

**Characteristics**	**All (*n* = 48)**	**Tenofovir (*n* = 24)**	**Placebo (*n* = 24)**	***p* value**
Rural % (*n*)	58.3% (28)	58.3% (14)	58.3% (14)	ns
Median age in years (IQR)	23 (22–25)	24 (22–28)	22 (22–23)	ns
Median days PI at enrolment (IQR)	38 (24–65)	35 (27–63)	45 (23–65)	ns
Median CD4 count (cells/μl) (IQR)	498 (434–655)	468 (444–569)	515 (433–685)	ns
Median viral load (copies/ml) (IQR)	59,0505	80,600	54.800	ns
	(17,300–135,500)	(22,000–130,000)	(13,600–148,000)	ns
Time to HIV infection from enrolment in months (IQR)	9.2 (4.9–14.1)	12.8 (6.6–16.6)	7.4 (3.3–10.6)	**0.02**
Completed high school % (*n*)	54.2% (26)	41.7% (10)	66.7% (16)	ns
Hormonal contraceptive use^a^ % (*n*)	97.9% (47)	100% (24)	95.8% (23)	ns
Marital Status % (*n*)				
Stable/married partner	79.2% (38)	87.5% (21)	70.8 (17)	ns
Single	18.7% (9)	12.5% (3)	25.0% (6)	ns
>2 partners	2.1% (1)	0.0% (0)	4.2% (1)	ns
Numbers of reported sexual partners in the last 3 months % (*n*)
0 to 1	93.8% (45)	95.8% (23)	91.7% (22)^b^	ns
2 to 5	6.2% (3)	4.2% (1)	8.3% (2)	ns
Reported condom used at last sex act % (*n*)	68.8% (33)	75.0% (18)	62.5% (15)	ns

*IQR, interquartile range; PI, post-infection*.

*Significant values were defined as p < 0.05 and indicated in bold text*.

a *Hormonal contraception use included the injectable norethisterone and depomedroxyprogesterone acetate, and oral contraception. One woman in the placebo arm of the study was using an intrauterine device*.

b *Two non-rapid progressing women had missing sexual partner data (in previous 3 months) as they refused to answer the question*.

### Gp41- and p24-Specific ADNP Increased Significantly in the GT of Women in the Tenofovir Compared to the Placebo Arm

**Figure 3:**
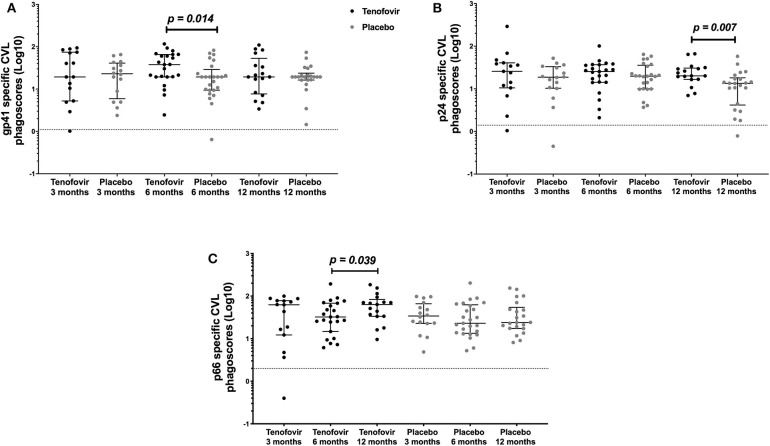
HIV-specific ADNP [phagoscores (Log10)] in the genital tract (CVL) in women from the tenofovir and placebo arms. Cross-sectional analyses between the tenofovir and placebo arms to HIV proteins **(A)** gp41 and **(B)** p24 and longitudinal analyses for tenofovir and placebo **(C)** p66. Solid lines indicate the minimum and maximum values of box and whisker plots, with black solid lines across the dots indicate the median at each time point for each study arm. Wilcoxon signed-rank test was used to analyse ADNP activity over time and Wilcoxon-Mann-Whitney test was used for cross sectional analysis. Statistically significant values were defined as p < 0.05 with no adjustments for multiple comparisons.

In order to determine if topical tenofovir had an effect on the ADNP responses in the GT, we compared HIV-specific ADNP responses at 3, 6, and 12 months to those in the placebo arm. GT gp41-specific ADNP in the tenofovir arm were significantly higher at 6 months [median and interquartile range (IQR)] (37.92; IQR = 19.40–65.60) compared to those in the placebo arm (19.40; IQR = 9.43–28.93) (p = 0.014) (Figure 3A). In addition, p24-specific ADNP were significantly higher in the tenofovir arm at 12 months (20.15; IQR = 16.61–30.61) compared to the placebo arm (13.41; IQR = 4.16–18.11) (p = 0.007) (Figure 3B). P66-specific ADNP trended higher in the tenofovir arm (63.58; IQR = 33.48–83.09) compared to the placebo arm (24.03; IQR = 17.44–54.16) at 12 months (p = 0.063, Supplementary Table 1). No significant differences were found between the arms, at any of the other time points, for gp120-specific ADNP (Supplementary Table 1).

In order to determine if there were changes in HIV-specific ADNP over time, we investigated these responses longitudinally in the GT in both arms. In the tenofovir arm, only p66-specific ADNP in the GT increased significantly from 6 to 12 months [IQR of 6 months (32.33; IQR = 14.68–67.99); 12 months (63.58; IQR = 33.48–83.09)] (p = 0.039) (Figure 3C). In contrast, the p66-specific ADNP in the placebo arm remained similar from 3 to 12 months. In the GT, within the arms, gp120-, gp41-, and p24-specific ADNP remained similar across all time points (Supplementary Table 1).

### Gp120- and p66-Specific ADNP Activities Increased in the Blood Over Time, Irrespective of Prior Topical Tenofovir Gel Use

**Figure 4:**
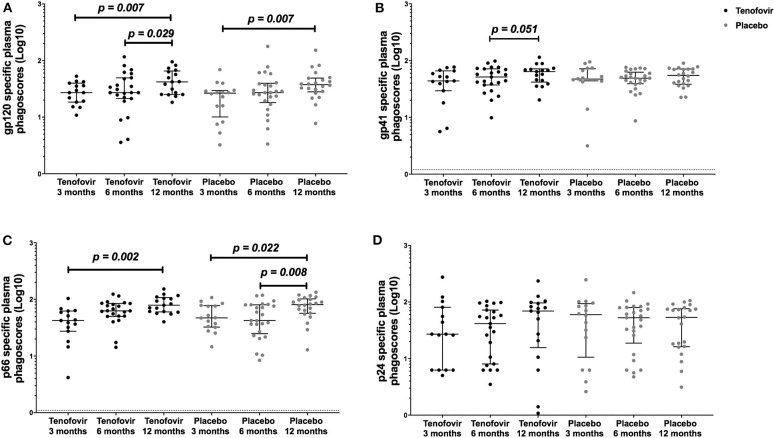
Longitudinal analyses of plasma HIV-specific ADNP [phagoscores (Log10)] in both the tenofovir and the placebo arms. Phagocytic activities [phagoscores (Log10)] to HIV proteins **(A)** gp120, **(B)** gp41, **(C)** p66, and **(D)** p24. Solid lines indicate the minimum and maximum values of box and whisker plots, with black solid lines across the dots indicate the median at each time point for each study arm. Statistical differences were defined as p < 0.05 and were determined by Wilcoxon signed-rank test. No adjustments were made for multiple comparisons.

Next, we aimed to understand the kinetics of the HIV-specific ADNP in the blood within each of the arms over time. Longitudinal analyses showed that in the tenofovir arm, gp120-specific ADNP consistently and significantly increased from 3 months (27.12; IQR = 18.49–39.85) to 12 months (41.99; IQR = 25.17–65.51) (p = 0.007) (Figure 4A) and from 6 (27.12; IQR = 21.32–49.47) to 12 months (41.99; IQR = 25.17–65.51) (p = 0.029). Similarly, a significant increase in gp120 ADNP was also observed in the placebo arm from 3 months (26.52; IQR = 10.08–29.56) to 12 months (37.52; IQR = 28.00–49.11, p = 0.007, Figure 4A). Gp41-specific ADNP in the tenofovir arm trended to increase from 6 months (51.52; IQR = 37.26–71.69) to 12 months, (64.10; IQR = 41.91–71.59) (p = 0.051, Figure 4B). In the tenofovir arm, p66-ADNP significantly increased from 3 months (42.35; IQR = 27.40–62.37) to 12 months (79.01; IQR = 60.21–107.50) (p = 0.002, Figure 4C). Similarly, in the placebo arm p66-specific ADNP significantly increased from 3 months (47.12; IQR = 32.40–77.85) to 12 months (80.89; IQR = 56.81–101.90) (p = 0.022, Figure 4C) and also from 6 months (42.41; IQR = 24.87–80.24) to 12 months (80.89; IQR = 56.81–101.90) (p = 0.008, Figure 4C). For p24-specific ADNP no significant differences were observed over time within the tenofovir or placebo arms (Figure 4D).

### P66-Specific ADNP Correlated Inversely Between the GT and Blood in the Placebo Arm

In order to determine if the magnitudes of ADNP activities in the blood mirrored those in the GT, cross-compartmental correlation analyses for both arms were conducted. In the placebo arm, only the p66-specific ADNP correlated inversely between the plasma and GT at 12 months post-infection (r = −0.48; p = 0.024, Supplementary Table 2). No other significant cross-compartment associations were found (Supplementary Table 2).

### Gp41-Specific ADCC Were Significantly Higher in the Genital Tract in the Tenofovir Arm

**Figure 5:**
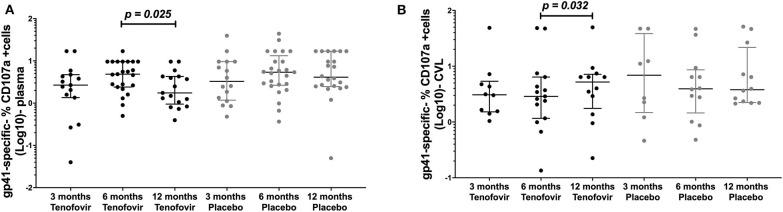
Longitudinal analyses of NK cell activated ADCC (antibody-specific- %CD107a^+^ cells [Log10]) in the plasma and GT (CVL) of women at 3 months, 6 months and 12 months, for the tenofovir and placebo arms for gp41. Analyses for cytotoxic activities (gp41-specific %CD107a^+^ cells [Log10]) in the plasma on the left **(A)** for gp41 and the genital tract on the right to **(B)** for gp41. Solid lines indicate the minimum and maximum values of box and whisker plots, with black solid lines across the dots indicate the median at each time point for each study arm. Wilcoxon signed-rank tests were done to determine statistical differences between NK cell activated ADCC activities within each arm of the study, for each protein. Statistically significant values were defined as p < 0.05. No adjustments were made for multiple comparisons.

Antibody mediated NK cell activation was measured by the % expression of CD107a positive (+) cells as a surrogate for ADCC. Longitudinal analyses in the tenofovir arm, showed that plasma gp41-specific ADCC significantly decreased at 12 months (1.75; IQR = 0.95–4.28) from the 6 month post-infection time-point (4.85; IQR = 2.40–9.60), (p = 0.025, Figure 5A). However, in the GT and in the tenofovir arm gp41-specific ADCC significantly increased at 12 months (5.20; IQR = 1.76–7.16) from 6 months post-infection (2.88; IQR = 1.17–6.39), (p = 0.032, Figure 5B). Plasma and GT gp120-, p66- or p24-specific ADCC were not different over time within each of the study arms (Supplementary Tables 3, 4).

### Gp41 and p24-Specific ADCC Responses in the Plasma Were Greater at Particular Time Points, in the Placebo Arm

We then assessed whether there were any differences for ADCC between the arms in the plasma and GT. We report significant differences in the plasma but not in the GT (Supplementary Table 4). Plasma HIV-gp41 ADCC responses in the placebo arm 4.11 (IQR = 2.47–16.90), were significantly higher at 12 months compared to the tenofovir arm 1.75 (IQR = 0.95–4.28), p = 0.017) (Supplementary Table 3). In the placebo arm, plasma p24-specific responses at 3 months were greater 3.80 (IQR = 2.55–7.23), than those in the tenofovir arm 1.75 (IQR = 1.25–4.54, p = 0.027) (Supplementary Table 3). Gp120- and p66-specific ADCC in both the tenofovir and placebo arms were similar for all timepoints (Supplementary Table 3). In the absence of further differences between the arms for any of the remaining HIV-specific ADCC activities in the plasma, we then sought to determine if there was concordance between HIV-specific ADCC in the plasma and GT in both arms at 3, 6, and 12 months.

### Gp41-Specific ADCC Responses in the Tenofovir Arm Correlated Between the Plasma and GT

To determine if HIV-specific ADCC in the blood mirrored those in the GT, cross-compartmental correlation analyses for both arms were conducted. At 6 months post-infection, gp41-specific ADCC in the plasma significantly and directly correlated with those in the GT of women in the tenofovir arm (r = 0.53; p = 0.045) (Table 2). No other significant correlations were observed for gp120, p66, or p24.

**Table 2:** Inter-compartmental correlations for the blood and genital tract for HIV-specific ADCC activities in the tenofovir and placebo arms.

**Protein**	**Arm**	**3 months**		**6 months**		**12 months**	
		**r-value**	**p-value**	**r-value**	**p-value**	**r-value**	**p-value**
gp120	Tenofovir	−0.15	0.708	−0.24	0.44	0.20	0.917
	Placebo	0.03	0.936	0.50	0.126	−0.15	0.653
gp41	Tenofovir	−0.27	0.441	**0.53**	**0.045**	−0.11	0.727
	Placebo	−0.39	0.337	0.45	0.138	−0.03	0.952
p66	Tenofovir	−0.17	0.800	−0.08	>0.999	0.08	0.818
	Placebo	0.41	0.571	0.58	0.250	−0.25	0.528
p24	Tenofovir	0.26	>0.999	−0.13	0.754	0.61	0.286
	Placebo	−0.35	0.800	−0.41	0.571	0.61	0.286

*Significant values were defined as p < 0.05 and indicated in bold text*.

### Plasma HIV-gp41 and p66-Specific ADNP Correlated to ADCC Activities in the Tenofovir Arm

**Figure 6:**
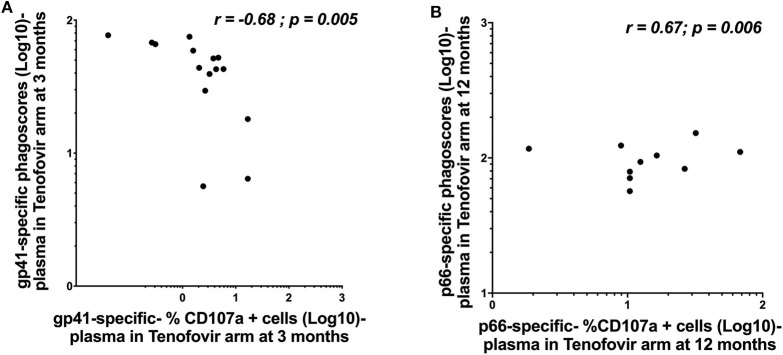
Correlations between ADNP (Log 10 Phagoscores) and NK cell activated ADCC (antibody-specific- %CD107a^+^ cells [Log10]) activities in the plasma of women in the tenofovir arm. **(A)** gp41-specific- phagoscores (Log10) at 3 months in the tenofovir arm, **(B)** p66-specific- %CD107a^+^ cells (Log10) at 12 months. Spearman R correlation analyses were performed. Significant values were identified as *p* < 0.05.

Whether HIV-specific antibodies mediating ADNP and ADCC occupy the same immunological space and can correspond in terms of magnitude remains less well defined. We, therefore, sought to determine if ADNP and ADCC activities correlated with each other within each of the compartments. In the tenofovir arm, gp41-specific ADNP inversely correlated with gp41-specific ADCC activities in the plasma at 3 months (r = −0.68; p = 0.005) (Figure 6A). Plasma p66-specific ADNP and ADCC correlated significantly at 12 months (r = 0.67; p = 0.006) in the tenofovir arm (Figure 6B). No correlations were observed for gp120 or p24-specific activities in the plasma. Additional linear mixed effects models were performed to also determine if these correlations were predictive of the effect of tenofovir on specific nNAb functions over time. These analyses yielded no significant findings (data not shown).

### Fc-Mediated ADCC and ADNP Correlated Inversely With gp120- and gp41 Specific Antibody Titres in the Tenofovir Arm Only

Next, we investigated whether the titres of the HIV-specific antibodies from our previous study of this cohort [Log10 (MFI ^*^dilution factor)] (14), directly correlated with the magnitude of ADNP and ADCC found in this study. In the tenofovir arm, significant inverse correlations were observed between the plasma gp41-specific ADNP and gp41-specific antibody titres at 6 months post-infection (r = −0.50; p = 0.015, Supplementary Table 5). Gp120-specific antibody titres in the tenofovir arm inversely correlated with gp120-specific ADCC in the plasma (r = −0.54; p = 0.009) (Supplementary Table 6) at 6 months. No further correlations for ADNP or ADCC were observed in relation to any of the other HIV-specific antibody titres in either compartments or study arms (Supplementary Tables 5, 6).

### V1V2-gp70-Specific ADNP and p24-Specific ADCC Correlated With CD4^+^ T Cell Counts in the Tenofovir Arm

**Figure 7:**
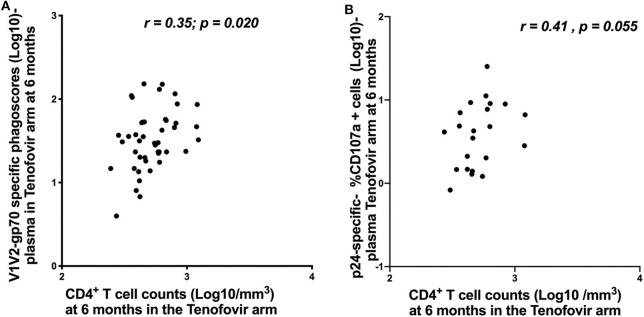
Correlations between CD4^+^ T cell counts and ADNP (Log 10 Phagoscores) and NK cell activated ADCC in the plasma for the tenofovir arm at 6 months. **(A)** V1V2-gp70-specific phagoscores (Log10) correlation with CD4^+^ T cell counts and **(B)** p24-specific- %CD107a^+^ cells (Log10]) correlation with CD4^+^ T cell counts. Significant values were identified as *p* < 0.05.

In order to understand the impact of nNAb functions on disease progression, we investigated the relationship between HIV-specific ADNP/ADCC activities and CD4^+^ T cell counts and viral loads. In the tenofovir arm at 6 months post-infection, V1V2-gp70-specific ADNP and p24-specific ADCC activities in the plasma directly correlated with CD4^+^ T cell counts [r = 0.35; p = 0.020—Figure 7A and r = 0.41; p = 0.055—Figure 7B, respectively], and no further associations were found.

## Discussion

Previous findings by our group demonstrated that topical PrEP use modulated HIV-1 antibody avidity post-seroconversion (7) underscoring potential public health implications for confirming incident HIV infections. Subsequently we showed higher detectability and titres of particular HIV-specific antibodies (14) in the genital tracts and blood of women who used the tenofovir gel prior to HIV-1 infection compared to those of the placebo gel users. We tested the hypothesis that the higher detectability and titres showed a corresponding increase in HIV-specific antibody Fc-mediated ADCC and ADNP functions in the genital tracts and the blood despite the reduced HIV-1 binding avidity (7) associated with tenofovir gel. We found gp120-and gp41-specific Fc-mediated antiviral activities in both compartments, regardless of prior topical tenofovir gel use.

HIV-specific antibodies from the genital tract and blood displayed nNAb functionality despite a prior study from our group having demonstrated that topical PrEP modulated plasma antibody binding avidity in these seroconverters (7). Although the HIV-specific nNAb activities were mostly similar between the tenofovir and placebo gel users, there were a few differences that distinguished the tenofovir from the placebo arm during the first 12 months post-infection. Despite gp41- and p24-specific ADNP in the blood being higher in the placebo compared to the tenofovir arm, genital tract gp41- and p24-specific ADNP were significantly higher in the tenofovir compared to the placebo arm at 6 and 12 months post-infection. The magnitudes of gp41- and p24-specific ADNP in the genital tract were also mirrored in the plasma. These data suggest the preservation of immune responses/function (8) in both compartments at least in the primary phase of HIV infection in women who used tenofovir. NNAbs isolated from vaginal samples of HIV infected women and more specifically gp41-specific IgG3 were shown to facilitate the phagocytosis of infectious HIV virions (33). The significantly increased p66-specific ADNP over time post-infection in the genital tract in the tenofovir arm, may be indicative of a developing immune response (11). Our data suggest that the significant delay in time to infection in the women who used tenofovir, an inhibitor of HIV reverse transcriptase (comprised of p66/p51 subunits) may have allowed for priming of p66-specific antibody immunity leading to the higher titres (14), and magnitude of ADNP over time. The functional significance of p66-specific antibodies is more challenging as p66 is not usually expressed on the surfaces of infected cells (13). P66-specific antibodies are likely elicited through the antigenic stimulation of infected cells undergoing subsequent cell lysis. Pol proteins may also be scavenged by antigen presenting cells, which then present these peptides subsequently targeted for degradation through ADNP or ADCC (13). These data provide evidence at least *in vitro* for the potential use of conserved viral proteins such as Pol and Gag as vaccine immunogens that elicit HIV-specific antibodies (12, 34) able to augment diverse antiviral activities.

In the tenofovir arm only, and in the blood, we also found significant inverse associations between ADNP and ADCC for gp41, and direct associations between ADNP and ADCC for p66. With prior PrEP use, these functions may be differentially regulated peripherally, as we found no such correlations between the two antiviral functions in the genital tract. Our data suggests that various nNAb functions may occupy specific compartmental spaces in pathogenic control, possibly to avoid redundancies. Whether both ADCC and ADNP need to be equally exerted in the genital tract and systemically to control infection, remains unknown.

We previously showed that HIV-specific IgG titres in the genital tract were considerably lower than those in the plasma in women from this study (14), the amount of antibodies in the genital tract depends on a combination of local IgG production (35) and transudation across the epithelial cell layer (36). Gp120-specific IgG titres inversely correlated with gp120-specific ADCC in the plasma, of women in the tenofovir arm. Env gp41-specific antibody titres also correlated inversely with gp41-specific ADNP. NHP studies have shown a strong and direct association between the Env IgG titres and ADCC function (in Env prime boost strategies) which protected against SHIV infection in the blood, vaginal and rectal mucosae (37, 38). Our data argue that the quality and not necessarily the quantity of these antibodies may be paramount in eliciting effective nNAb and neutralizing antiviral functions (39–41) and are analogous to the immune correlate studies in the RV144 trial where the quality of antibody activities were suggested to be a better surrogate indicator of reduced risk of infection (17, 42–44).

Viral control in elite controllers (45), reductions in the genital tract viral loads (28, 29) and delayed HIV disease progression (46) have been associated with increased ADCC. V1V2-gp70 loop specific antibodies identified as immune correlates of reduced risk of infection in the RV144 trial vaccinees (17, 18, 47, 48) elicited ADCC, virion capture and limited tier 1 virus neutralization (49–51). P24-specific IgG1 responses were associated with reduced viral loads (10, 52, 53) and improved CD4^+^ T cell counts in HIV-1 subtype C infected participants (9). The direct associations between V1V2-gp70-specific ADNP, p24-specific ADCC and CD4^+^ T cell counts in the plasma of HIV infected women from the tenofovir arm suggest that poly-functional antibodies may be needed to synergise nNAb activities to not only control viral load (44) and impact HIV disease progression but also to confer protection. We show that in infected women exposed to a topical PrEP, genital tract gp41, and gp120-specific IgG are able to mediate diverse functional activities such as ADCC. In HIV uninfected women from the HPTN035 microbicide trial only gp120-specific IgA could be detected in the genital tract (17). Whether the gp120-specific IgA of women from the HPTN035 trial were able to effect functional activity remains unknown. Our data suggest that prior topical PrEP did not modulate gp120 or gp41-specific IgG Fc mediated ADCC and that PrEP may be used in parallel as an HIV prevention strategy with vaccination that aims to induce envelope antibodies (17). Although these nNAbs did not prevent these women from acquiring HIV in our study, antibodies directed toward V1V2-gp70, gp120 (10, 18, 44, 54) and p24 that elicit ADCC have been correlated with slower disease progression (9, 46) or elite control (55) and a functional immune response (8, 9).

One of the limitations of this study is that we showed limited differences in HIV-specific nNAb functions between the arms despite having previously shown that higher HIV-specific antibody titres and detection associated with tenofovir gel (14). Indeed, compared to topical PrEP, oral PrEP may have a markedly different impact on immune responses. The lack of more distinct differences between the arms may in part be attributed to just over half of the tenofovir gel users, having detectable tenofovir levels in their CVLs likely indicative of women using the tenofovir gel inconsistently or not at all [reviewed by Baxter and Abdool Karim (56)]. Alternatively, tenofovir levels were dependent on women anticipating or having coitus with their partners which may or may not have coincided with the follow-up clinic visits where specimen collection was performed. It should be noted that these tenofovir levels in the genital tract were measured prior to HIV infection, and once women tested positive for HIV, the use of the topical tenofovir gel was discontinued. Furthermore, limited volumes of genital samples available, and the low concentrations of IgGs inherent in the CVL samples through dilution precluded further enrichment and verification of the specific IgG subclass or subclasses that mediated the ADCC activities (28). Antibodies from these diluted genital tract specimens contain a heterogenous pool of immunoglobulins which may have modulated or interfered with nNAb function in contrast to the purified IgG from the plasma specimens. Additionally, we further acknowledge that differences in the concentrations in the IgGs between the plasma and GT precluded comparisons for the nNAb functions between the compartments. Despite the shortcomings of the CVL specimens with the lower antibody concentrations and other factors that may interfere with nNAb functions than the plasma purified IgG, the CVL does represent more accurately the milieu *in vivo* and contains antibodies that facilitate antiviral functions. This is more especially for gp-120 specific antibodies in the genital tract that despite having lower titres (14), still exerted ADNP and ADCC. A further limitation is that we did not evaluate the impact of HIV-specific IgA-mediated nNAb activities in the blood or genital tracts of women. Env-specific IgAs in the blood have been shown to block ADCC activities mediated by IgG (57) whereas gp41-specific IgAs inhibited HIV-viral transcytosis across the mucosal tissue (58), mediated phagocytosis and blocked HIV-1 binding to host cell receptors (59). The contribution of IgAs on IgG mediated effector functions in the genital tract requires further investigation. Together these data further emphasize that in high risk populations where combination prevention strategies are required to prevent HIV, antibodies capable of diverse antiviral functions can be elicited at mucosal portals of entry.

## Conclusions

The limited differences in HIV-specific nNAb functions between the tenofovir and placebo arms suggest that prior topical PrEP did not modulate humoral immune functions post HIV-seroconversion. Moreover, the simultaneous assessment of these two nNAb functions in both the compartments allowed us to gauge the discrepant and common HIV antibody-specific antiviral activities in different immunological spaces. These nNAb functions may be compartment-specific possibly to avoid immunological redundancies or to avert extra inflammation associated with nNAb-mediated activities. Together, the data provide further evidence for the role of nNAbs as quintessential to a diverse functional immune profile, especially in the area of vulnerability, the female genital tract.

## Data Availability Statement

The datasets generated for this study are available on request to the corresponding author.

## Ethics Statement

The studies involving human participants were reviewed and approved by University of KwaZulu-Natal Biomedical Research Ethics Committee. The patients/participants provided their written informed consent to participate in this study.

## Author Contributions

KF designed and ran experiments, acquired and analyzed the data, and wrote and edited the manuscript. DA conceptualized, designed and acquired funding for the study, and wrote and edited the manuscript with CB. JM, BN, and DA assisted in study design. JM and AS assisted in experiments and JM and FO assisted in and verified the data analysis. DA, JM, SN, QA, and SA provided laboratory space for experimental procedures, samples to run the experiments, and edited the manuscript along with J-AP and AC. All authors contributed to the article and approved the submitted version.

## Conflict of Interest

The authors declare that the research was conducted in the absence of any commercial or financial relationships that could be construed as a potential conflict of interest. The reviewer JP declared a past co-authorship with one of the authors SA to the handling editor.
