# Supplementary material for: Topical Tenofovir Pre-exposure Prophylaxis and Mucosal HIV-Specific Fc-Mediated Antibody Activities in Women
Source: Front Immunol. 2020 Jul 6;11:1274. doi: 10.3389/fimmu.2020.01274 (PMC7357346; doi:10.3389/fimmu.2020.01274)
Supplement: Supplementary file 1 [file Table_1.DOCX]

| Supplementary Table 1: Comparison of ADNP activities between the tenofovir and placebo arms for gp120, gp41, p66 and p24 at 3, 6 and 12 months in the GT and plasma | | | | | | | | | | |
| --- | --- | --- | --- | --- | --- | --- | --- | --- | --- | --- |
| ADNP Genital tract responses | | | | | | | | | | |
|  | **3 months** | | | **6 months** | | | | **12 months** | | |
|  | **Tenofovir** | **Placebo** | **p-value** | **Tenofovir** | **Placebo** | **p-value** | | **Tenofovir** | **Placebo** | **p-value** |
| HIV protein | **Median (IQR)** | |  | **Median (IQR)** | | |  | **Median (IQR)** | |  |
| Gp120 | 9.95  (3.83 – 14.72) | 9.54  (7.30 – 20.35) | 0.572 | 11.81  (6.07 – 21.10) | 9.73  (3.43 – 14.23) | 0.235 | | 9.50  (4.12- 19.44) | 11.21  (6.71 – 18.45) | 0.721 |
| Gp41 | 19.40  (5.24 – 74.55) | 23.04  (5.94 – 41.09) | 0.579 | 37.92  (19.40 – 65.60) | 19.40  (9.43 – 28.93) | **0.014** | | 19.40  (7.69 – 53.40) | 19.40  (16.39 – 23.75) | 0.642 |
| P66 | 62.56  (12.24 – 78.28) | 34.01  (22.82 – 65.93) | 0.892 | 32.33  (14.68 – 67.99) | 22.98  (13.37 – 62.46) | 0.570 | | 63.58  (33.48 – 83.09) | 24.03  (17.44 – 54.16) | **0.063** |
| P24 | 25.67  (10.54 – 40.76) | 18.71  (10.32 – 33.13) | 0.358 | 25.40  (14.20 – 36.81) | 19.81  (9.99 – 35.65) | 0.461 | | 20.15  (16.61 – 30.61) | 13.41  (4.16 – 18.11) | **0.007** |
| ADNP Plasma response | | | | | | | | | | |
|  | **3 months** |  |  | **6 months** |  |  | | **12 months** |  |  |
|  | **Tenofovir** | **Placebo** | **p-value** | **Tenofovir** | **Placebo** | **p-value** | | **Tenofovir** | **Placebo** | **p-value** |
| HIV protein | **Median (IQR)** |  |  | **Median (IQR)** |  |  | | **Median (IQR)** |  |  |
| Gp120 | 27.12  (18.49-39.85) | 26.52  (10.08 – 29.56) | 0.295 | 27.12  (21.32 – 49.47) | 27.12  (18.14 – 39.65) | 0.759 | | 41.99  (25.17 – 65.51) | 37.52  (28.00-49.11) | 0.664 |
| Gp41 | 43.85  (29.48 – 66.04) | 47.26  (44.08 – 71.82) | 0.446 | 51.52  (37.26 – 71.69) | 49.00  (39.18 – 62.56) | 0.759 | | 64.10  (41.91 – 71.59) | 54.74  (38.35 – 71.13) | 0.644 |
| P66 | 42.35  (27.40 – 62.37) | 47.12  (32.40 – 77.85) | 0.338 | 62.76  (50.15 – 84.58) | 42.41  (24.87 – 80.24) | 0.210 | | 79.01  (60.21 – 107.50) | 80.89  (56.81 – 101.90) | 0.664 |
| P24 | 27.01  (6.26 – 80.82) | 59.78  (10.59 – 93.42) | 0.512 | 41.51  (8.02 – 71.99) | 52.79  (18.71 – 79.68) | 0.609 | | 69.16  (15.63 – 96.35) | 53.48  (16.24 – 76.11) | 0.457 |
