# Supplementary material for: Topical Tenofovir Pre-exposure Prophylaxis and Mucosal HIV-Specific Fc-Mediated Antibody Activities in Women
Source: Front Immunol. 2020 Jul 6;11:1274. doi: 10.3389/fimmu.2020.01274 (PMC7357346; doi:10.3389/fimmu.2020.01274)
Supplement: Supplementary file 2 [file Table_2.docx]

| Supplementary Table 2: Compartmental correlations of phagocytic activities (ADNP) between the plasma and the GTs (CVL) of women, in the Tenofovir and Placebo arms, for all HIV-proteins | | | | | | | |
| --- | --- | --- | --- | --- | --- | --- | --- |
| Protein | **Arm** | **3 months** | | **6 months** | | **12 months** | |
|  |  | *r-value* | *p-value* | *r-value* | *p-value* | *r-value* | *p-value* |
| gp120 | Tenofovir | -0.03 | 0.905 | 0.35 | 0.099 | 0.01 | 0.966 |
|  | *Placebo* | -0.27 | 0.313 | 0.05 | 0.822 | -0.37 | 0.092 |
| gp41 | Tenofovir | 0.45 | 0.092 | -0.20 | 0.372 | 0.004 | 0.991 |
|  | *Placebo* | 0.43 | 0.096 | -0.06 | 0.769 | 0.26 | 0.245 |
| p66 | Tenofovir | 0.05 | 0.873 | 0.26 | 0.231 | -0.30 | 0.246 |
|  | *Placebo* | -0.15 | 0.586 | -0.26 | 0.215 | **-0.48** | **0.024** |
| p24 | Tenofovir | 0.09 | 0.753 | -0.34 | 0.113 | -0.17 | 0.509 |
|  | *Placebo* | -0.17 | 0.536 | 0.03 | 0.887 | -0.05 | 0.817 |
| significant values defined as p<0.05 are indicated in bold text | | | | | | | |
