# Supplementary material for: Topical Tenofovir Pre-exposure Prophylaxis and Mucosal HIV-Specific Fc-Mediated Antibody Activities in Women
Source: Front Immunol. 2020 Jul 6;11:1274. doi: 10.3389/fimmu.2020.01274 (PMC7357346; doi:10.3389/fimmu.2020.01274)
Supplement: Supplementary file 3 [file Table_3.DOCX]

| **Supplementary Table 3: Cross-sectional analyses of plasma ADCC activities mediated by IgG from women in the tenofovir and placebo arms, at 3, 6 and 12 months, post-infection** | | | | | | | | | |
| --- | --- | --- | --- | --- | --- | --- | --- | --- | --- |
| **3 months** | | | | | | | | | |
|  | **%CD107a** | **%CD107a** |  | **%IFN-γ** | **%IFN-γ** |  | **%MIP-1β** | **%MIP-1β** |  |
|  | **Tenofovir** | **Placebo** | **p-value** | **Tenofovir** | **Placebo** | **p-value** | **Tenofovir** | **Placebo** | **p-value** |
|  | **Median (IQR)** | **Median (IQR)** |  | **Median (IQR)** | **Median (IQR)** |  | **Median (IQR)** | **Median (IQR)** |  |
|  | **n=15** | **n=16** |  | **n=15** | **n=16** |  | **n=15** | **n=16** |  |
| gp120 | 1.12 (1.08-2.07) | 1.12 (1.12-3.80) | 0.365 | 20.36 (12.36-21.16) | 12.36 (12.36-21.16) | 0.189 | 14.67 (4.20-15.38) | 13.33 (9.42-16.33) | 0.484 |
|  |  |  |  |  |  |  |  |  |  |
| gp41 | 2.68 (1.35-4.71) | 3.27 (1.18-9.60) | 0.526 | 2.06 (1.30-6.38) | 1.86 (0.73-32.32) | 0.777 | 23.37 (4.03-75.06) | 16.15 (5.49-75.06) | 0.684 |
|  |  |  |  |  |  |  |  |  |  |
| p66 | 10.40 (0.20-38.15) | 8.80 (2.84-12.70) | 0.894 | 1.84 (1.84-6.55) | 1.84 (1.84-5.79) | 0.500 | 27.08 (25.17-33.30) | 27.08 (25.15-30.02) | 0.483 |
|  |  |  |  |  |  |  |  |  |  |
| p24 | 1.75 (1.25-4.54) | 3.80 (2.55-7.23) | **0.027** | 3.44 (2.27-5.09) | 3.53 (2.29 -8.48) | 0.830 | 18.95 (14.00-25.20) | 20.50 (17.20-26.90) | 0.513 |
|  |  |  |  |  |  |  |  |  |  |
| **6 months** | | | | | | | | | |
|  | **%CD107a** | **%CD107a** |  | **%IFN-γ** | **%IFN-γ** |  | **%MIP-1β** | **%MIP-1β** |  |
|  | **Tenofovir** | **Placebo** | **p-value** | **Tenofovir** | **Placebo** | **p-value** | **Tenofovir** | **Placebo** | **p-value** |
|  | **Median (IQR)** | **Median (IQR)** |  | **Median (IQR)** | **Median (IQR)** |  | **Median (IQR)** | **Median (IQR)** |  |
|  | **n=23** | **n=25** |  | **n=23** | **n=25** |  | **n=23** | **n=25** |  |
| gp120 | 1.16 (1.12-3.75) | 1.85 (1.12-3.80) | 0.517 | 12.36 (5.07-21.36) | 12.36 (4.25-21.16) | 0.729 | 7.38 (2.90-14.67) | 12.83 (7.34-15.10) | 0.181 |
|  |  |  |  |  |  |  |  |  |  |
| gp41 | 4.85 (2.40-9.60) | 5.38 (2.65-13.25) | 0.604 | 1.94 (1.15-12.79) | 2.03 (0.51-19.68) | 0.583 | 23.37 (3.63-75.06) | 23.37 (8.93-75.06) | 0.307 |
|  |  |  |  |  |  |  |  |  |  |
| p66 | 8.74 (0.47-27.55) | 10.40 (2.86-19.96) | 0.731 | 1.84 (1.84-6.11) | 1.84 (1.84-5.41) | 0.735 | 27.08 (18.83-33.30) | 27.57 (27.08-33.30) | 0.713 |
|  |  |  |  |  |  |  |  |  |  |
| p24 | 4.26 (1.47-8.69) | 3.08 (2.26-4.26) | 0.428 | 4.06 (2.42-6.97) | 3.64 (1.33-6.40) | 0.693 | 25.00 (14.90-30.70) | 21.63 (19.05-25.73) | 0.444 |
|  |  |  |  |  |  |  |  |  |  |
| **12 months** | | | | | | | | | |
|  | **%CD107a** | **%CD107a** |  | **%IFN-γ** | **%IFN-γ** |  | **%MIP-1β** | **%MIP-1β** |  |
|  | **Tenofovir** | **Placebo** | **p-value** | **Tenofovir** | **Placebo** | **p-value** | **Tenofovir** | **Placebo** | **p-value** |
|  | **Median (IQR)** | **Median (IQR)** |  | **Median (IQR)** | **Median (IQR)** |  | **Median (IQR** | **Median (IQR)** |  |
|  | **n=17** | **n=22** |  | **n=17** | **n=22** |  | **n=17** | **n=22** |  |
| gp120 | 3.80 (0.85-7.96) | 1.82 (0.57-4.07) | 0.233 | 7.19 (1.14-22.38) | 15.03 (6.55-21.16) | 0.164 | 9.00 (3.66-13.28) | 13.55 (7.55-17.27) | **0.053** |
|  |  |  |  |  |  |  |  |  |  |
| gp41 | 1.75 (0.95-4.28) | 4.11 (2.47-16.90) | **0.017** | 2.74 (1.42-18.47) | 2.58 (0.92-10.17) | 0.747 | 20.15 (3.83-75.06) | 23.75 (9.28-75.06) | 0.580 |
|  |  |  |  |  |  |  |  |  |  |
| p66 | 8.95 (0.37-14.45) | 10.40 (2.51-40.43) | 0.171 | 2.08 (1.84-6.38) | 1.84 (1.66-1.84) | 0.022 | 27.08 (16.25-33.30) | 33.27 (16.79-36.10) | 0.276 |
|  |  |  |  |  |  |  |  |  |  |
| p24 | 3.72 (2.04-7.16) | 3.23 (2.13-6.26) | >0.999 | 4.28 (2.49-7.94) | 3.08 (2.30-4.25) | 0.154 | 23.30 (18.63-29.80) | 22.95 (16.65-27.53) | 0.977 |
|  |  |  |  |  |  |  |  |  |  |
| *Significant differences were identified as p<0.05* | | | | | | | | | |
