# Supplementary material for: Topical Tenofovir Pre-exposure Prophylaxis and Mucosal HIV-Specific Fc-Mediated Antibody Activities in Women
Source: Front Immunol. 2020 Jul 6;11:1274. doi: 10.3389/fimmu.2020.01274 (PMC7357346; doi:10.3389/fimmu.2020.01274)
Supplement: Supplementary file 4 [file Table_4.DOCX]

| **Supplementary Table 4: Cross-sectional analyses of ADCC activities mediated by IgG from the genital tracts of women in the tenofovir and placebo arms, at 3, 6 and 12 months, post-infection** | | | | | | | | | | | | |
| --- | --- | --- | --- | --- | --- | --- | --- | --- | --- | --- | --- | --- |
| **3 months** | | | | | | | | | | | | |
|  | **%CD107a** | **%CD107a** |  | **%IFN-γ** | **%IFN-γ** | |  | **%MIP-1β** | **%MIP-1β** |  | | |
|  | **Tenofovir** | **Placebo** | **p-value** | **Tenofovir** | **Placebo** | | **p-value** | **Tenofovir** | **Placebo** | **p-value** | | |
|  | **Median (IQR)** | **Median (IQR)** |  | **Median (IQR)** | **Median (IQR)** | |  | **Median (IQR)** | **Median (IQR)** |  | | |
|  | **n=15** | **n=16** |  | **n=15** | **n=16** | |  | **n=15** | **n=16** |  | | |
| gp120 | 28.45 (14.85 -31.95) | 15.05 (3.70-45.50) | 0.545 | 1.33 (1.33-1.33) | 1.33 (1.08-1.33) | | 0.629 | 35.00 (20.70-47.70) | 26.10 (17.25-47.20) | 0.545 | | |
|  |  |  |  |  |  | |  |  |  |  | | |
| gp41 | 3.08 (1.52-5.37) | 6.89 (1.48-38.43) | 0.697 | 2.36 (2.36-2.36) | 2.36 (2.36-2.36) | | 0.523 | 20.85 (20.85-20.85) | 20.85 (20.85-33.64) | 0.330 | | |
|  |  |  |  |  |  | |  |  |  |  | | |
| p66 | 4.96 (4.96 -4.96) | 4.96 (4.96-4.96) | >0.99 | 0.50 (0.28-1.37) | 1.72 (1.16-5.52) | | **0.011** | 18.40 (3.58-18.40) | 18.40 (13.95-18.40) | 0.712 | | |
|  |  |  |  |  |  | |  |  |  |  | | |
| p24 | 2.15 (2.15-5.20) | 2.15 (2.15-2.84) | >0.99 | 8.08 (3.52-50.28) | 8.48 (3.52-46.13) | | 0.721 | 32.20 (5.40-32.20) | 32.20 (20.35-32.20) | >0.99 | | |
|  |  |  |  |  |  | |  |  |  |  | | |
| **6 months** | | | | | | | | | | | | |
|  | **%CD107a** | **%CD107a** |  | **%IFN-γ** | **%IFN-γ** | |  | **%MIP-1β** | **%MIP-1β** |  | | |
|  | **Tenofovir** | **Placebo** | **p-value** | **Tenofovir** | **Placebo** | | **p-value** | **Tenofovir** | **Placebo** | **p-value** | | |
|  | **Median (IQR)** | **Median (IQR)** |  | **Median (IQR)** | **Median (IQR)** | |  | **Median (IQR)** | **Median (IQR)** |  | | |
|  | **n=23** | **n=25** |  | **n=23** | **n=25** | |  | **n=23** | **n=25** |  | | |
| gp120 | 19.70 (7.35-31.28) | 27.95 (15.05-56.95) | 0.288 | 1.33 (1.33-1.33) | 1.33 (0.79-1.33) | | 0.422 | 33.00 (25.50-54.60) | 50.40 (33.00-60.90) | | | 0.270 |
|  |  |  |  |  |  | |  |  |  | | |  |
| gp41 | 2.88 (1.17-6.39) | 3.94 (1.45-8.62) | 0.548 | 2.36 (2.36-2.36) | 2.36 (2.36-2.36) | | 0.513 | 20.85 (20.85-20.85) | 20.85 (20.85-20.85) | | | >0.99 |
|  |  |  |  |  |  | |  |  |  | | |  |
| p66 | 4.96 (4.96-4.96) | 4.96 (0.74-4.96) | >0.99 | 0.74 (0.09-1.16) | 1.16 (0.55-1.68) | | 0.185 | 18.40 (18.40-18.40) | 18.40 (15.53-18.40) | | | 0.868 |
|  |  |  |  |  |  | |  |  |  | | |  |
| p24 | 2.15 (2.15-2.78) | 2.15 (2.15-2.15) | 0.387 | 27.08 (2.53-56.53) | 3.52 (3.43-5.12) | | 0.134 | 32.20 (19.95-32.20) | 13.85 (4.75-32.20) | | | 0.165 |
|  |  |  |  |  |  | |  |  |  |  | | |
| **12 months** | | | | | | | | | | | | |
|  | **%CD107a** | **%CD107a** |  | **%IFN-γ** | **%IFN-γ** | |  | **%MIP-1β** | **%MIP-1β** | |  | |
|  | **Tenofovir** | **Placebo** | **p-value** | **Tenofovir** | **Placebo** | **p-value** | | **Tenofovir** | **Placebo** | | **p-value** | |
|  | **Median (IQR)** | **Median (IQR)** |  | **Median (IQR)** | **Median (IQR)** |  | | **Median (IQR** | **Median (IQR)** | |  | |
|  | **n=17** | **n=22** |  | **n=17** | **n=22** |  | | **n=17** | **n=22** | |  | |
| gp120 | 15.05 (6.05-29.55) | 23.95 (9.40-31.00) | 0.743 | 1.33 (1.33-1.33) | 1.33 (0.73-1.33) | 0.633 | | 41.20 (26.60-51.00) | 36.50 (33.00-49.90) | | 0.853 | |
|  |  |  |  |  |  |  | |  |  | |  | |
| gp41 | 5.20 (1.76-7.16) | 3.79 (2.24-21.68) | 0.854 | 2.36 (2.36-2.36) | 2.36 (2.36-2.36) | 0.193 | | 20.85 (20.85-20.85) | 20.85 (20.85-20.85) | | >0.99 | |
|  |  |  |  |  |  |  | |  |  | |  | |
| p66 | 4.96 (4.96-4.96) | 4.96 (3.24-4.96) | 0.868 | 1.02 (0.41-1.16) | 1.16 (0.15-2.82) | 0.902 | | 18.40 (18.40-18.40) | 18.40 (18.40-18.40) | | >0.99 | |
|  |  |  |  |  |  |  | |  |  | |  | |
| p24 | 2.15 (2.15-2.15) | 2.15 (2.15-2.15) | >0.99 | 3.91 (2.85-36.03) | 3.52 (3.52-10.15) | 0.862 | | 25.00 (5.23-32.20) | 32.20 (16.75-32.20) | | 0.315 | |
|  |  |  |  |  |  | |  |  |  | |  | |
| *significant differences are identified as p<0.05* | | | | | | | | | | | | |
