# Supplementary material for: Topical Tenofovir Pre-exposure Prophylaxis and Mucosal HIV-Specific Fc-Mediated Antibody Activities in Women
Source: Front Immunol. 2020 Jul 6;11:1274. doi: 10.3389/fimmu.2020.01274 (PMC7357346; doi:10.3389/fimmu.2020.01274)
Supplement: Supplementary file 5 [file Table_5.DOCX]

| Supplementary Table 5: Correlations between the HIV-specific IgG titres and ADNP at 6 months post-infection, in the plasma and genital tract (CVL) | | | | | | | | |
| --- | --- | --- | --- | --- | --- | --- | --- | --- |
|  | | | | | | | | |
|  | **IgG titres (Log10)**  **vs plasma ADNP**  **(Tenofovir) n = 23** |  | **IgG titres (Log10)**  **vs plasma ADNP**  **(Placebo) n = 23** |  | **IgG titres (Log10)**  **vs CVL ADNP**  **(Tenofovir) n = 23** |  | **IgG titres (Log10)**  **vs CVL ADNP**  **(Placebo) n = 24** |  |
|  | ***r-value*** | ***p-value*** | ***r-value*** | ***p-value*** | ***r-value*** | ***p-value*** | ***r-value*** | ***p-value*** |
| gp120 | -0.03 | 0.907 | 0.10 | 0.659 | 0.10 | 0.635 | -0.07 | 0.750 |
| gp41 | **-0.50** | **0.015** | 0.02 | 0.942 | -0.08 | 0.705 | 0.15 | 0.484 |
| Gp70 | -0.03 | 0.883 | -0.11 | 0.606 | - | - | - | - |
| p66 | 0.26 | 0.227 | -0.40 | 0.062 | 0.05 | 0.824 | -0.18 | 0.407 |
| p24 | 0.23 | 0.283 | 0.32 | 0.147 | -0.23 | 0.325 | -0.24 | 0.272 |
