# Supplementary material for: Topical Tenofovir Pre-exposure Prophylaxis and Mucosal HIV-Specific Fc-Mediated Antibody Activities in Women
Source: Front Immunol. 2020 Jul 6;11:1274. doi: 10.3389/fimmu.2020.01274 (PMC7357346; doi:10.3389/fimmu.2020.01274)
Supplement: Supplementary file 6 [file Table_6.DOCX]

| Supplementary Table 6: Correlations between the HIV-specific IgG titres and the NK cell activated cytotoxic activities (ADCC) at 6 months post-infection, in the plasma and the GT (CVL) | | | | | | | | |
| --- | --- | --- | --- | --- | --- | --- | --- | --- |
|  | **IgG titres (Log10) vs plasma ADCC**  **- CD107a (Tenofovir) n = 23** | | **IgG titres (Log10) vs plasma ADCC**  **- CD107a (Placebo) n = 23** | | **IgG titres (Log10) vs CVL**  **ADCC**  **-CD107a (Tenofovir) n = 12** | | **IgG titres (Log10) vs CVL ADCC**  **- CD107a (Placebo) n = 10** | |
|  | ***r-value*** | ***p-value*** | ***r-value*** | ***p-value*** | ***r-value*** | ***p-value*** | ***r-value*** | ***p-value*** |
| gp120 | **-0.54** | **0.009** | -0.13 | 0.561 | -0.06 | 0.859 | 0.19 | 0.576 |
| gp41 | -0.26 | 0.238 | 0.04 | 0.857 | -0.33 | 0.231 | -0.02 | 0.950 |
| p66 | 0.28 | 0.206 | 0.32 | 0.144 | -0.20 | 0.727 | 0.02 | 0.967 |
| p24 | -0.34 | 0.313 | 0.24 | 0.289 | -0.18 | 0.724 | 0.25 | 0.750 |
